# Airborne bacteria in show caves from Southern Spain

**DOI:** 10.15698/mic2021.10.762

**Published:** 2021-07-26

**Authors:** Irene Dominguez-Moñino, Valme Jurado, Miguel Angel Rogerio-Candelera, Bernardo Hermosin, Cesareo Saiz-Jimenez

**Affiliations:** 1 Instituto de Recursos Naturales y Agrobiologia, IRNAS-CSIC, 41012 Sevilla, Spain.

**Keywords:** aerobiology, airborne bacteria, phototrophic biofilms, Bacillus, Arthrobacter, Micrococcus

## Abstract

This work presents a study on the airborne bacteria recorded in three Andalusian show caves, subjected to different managements. The main differences within the caves were the absence of lighting and phototrophic biofilms in *Cueva de Ardales*, the periodic maintenance and low occurrence of phototrophic biofilms in *Gruta de las Maravillas*, and the abundance of phototrophic biofilms in speleothems and walls in *Cueva del Tesoro*. These factors conditioned the diversity of bacteria in the caves and therefore there are large differences among the CFU m^-3^, determined using a suction impact collector, equipment widely used in aerobiological studies. The study of the bacterial diversity, inside and outside the caves, indicates that the air is mostly populated by bacteria thriving in the subterranean environment. In addition, the diversity seems to be related with the presence of abundant phototrophic biofilms, but not with the number of visitors received by each cave.

## INTRODUCTION

The region of Andalusia, Southern Spain, has an important number of show caves and shelters, most of them containing Paleolithic art and with high cultural interest. *Nerja, Tesoro, Piletas, Ardales* and *Murcielagos*, are some representative caves with rock art, receiving different numbers of visitors and adopting distinct conservation and management protocols [1]. Other caves are visited by its geological interest and the occurrence of characteristic speleothems, such as *Gruta de las Maravillas* [2].

One of the management practices, not frequently adopted in cave conservation, is a periodic aerobiological monitoring. In fact, the major threat for a show cave is derived from the presence and colonization of microorganisms in the air, water pools, sediments and speleothems. This is particularly important when bacteria and fungi threat the paintings. A few and well-known examples can be found in Altamira [3–5] and Lascaux caves [6–8].

To help in the cave management of airborne fungi, Porca *et al.* [9] launched an ecological indicator that provides data on the amount of fungal spores and marks the limits of danger for the conservation of Paleolithic paintings. These authors categorized caves into five classes, from one without fungal problems to the worst case, a cave with irreversible ecological disturbance. The two last classes generally corresponded to caves experiencing fungal outbreaks, phototrophic biofilms resulting from artificial lighting, or massive visits. The introduction of this ecological indicator was possible due to the well-characterized airborne fungal patterns, but unfortunately, this cannot be extended to bacteria. Indeed, bacteria do not present definite patterns in aerobiological studies, thus avoiding forecasting of bacterial outbreaks in caves. As a consequence, aerobiological studies should be adopted for each cave individually in order to measure airborne bacteria and adopt preventive conservation actions. The literature records several studies on bacteria in cave air [7, 10–16], but reports different conclusions.

The three show caves studied are located in Andalusia (Southern Spain) and were *Cueva del Tesoro, Cueva de Ardales* and *Gruta de las Maravillas. Cueva del Tesoro* and *Cueva de Ardales* contain valuable Paleolithic paintings and engravings, which can be observed by the visitors, while *Gruta de las Maravillas,* without paintings, holds rare speleothems. Comprehensive data on these caves were reported in a previous work investigating the aerobiological behavior of fungi [17]. The data previously reported include number of visitors at the sampling time, geomorphology and microclimatological data (temperature, concentration of carbon dioxide and radon) inside the caves. Briefly, in the sampling year the visits ranged from 130,314 peoples in *Gruta de las Maravillas* to 4,018 in *Cueva de Ardales*. *Cueva del Tesoro* received 28,257 visitors. Cave lengths were from about 1,500 to 2,130 m. Temperatures inside the caves ranged between 14.8°C and 17.4°C [17].

The main differences in the caves were the absence of lighting and phototrophic biofilms in *Cueva de Ardales*, the periodic maintenance and scarce occurrence of phototrophic biofilms in *Gruta de las Maravillas*, and the abundance of phototrophic biofilms in the speleothems and walls, in all the rooms and galleries, of *Cueva del Tesoro*. Cleaning and removal of biofilms were carried out in 2015, after this study [18].

**Figure 1:**
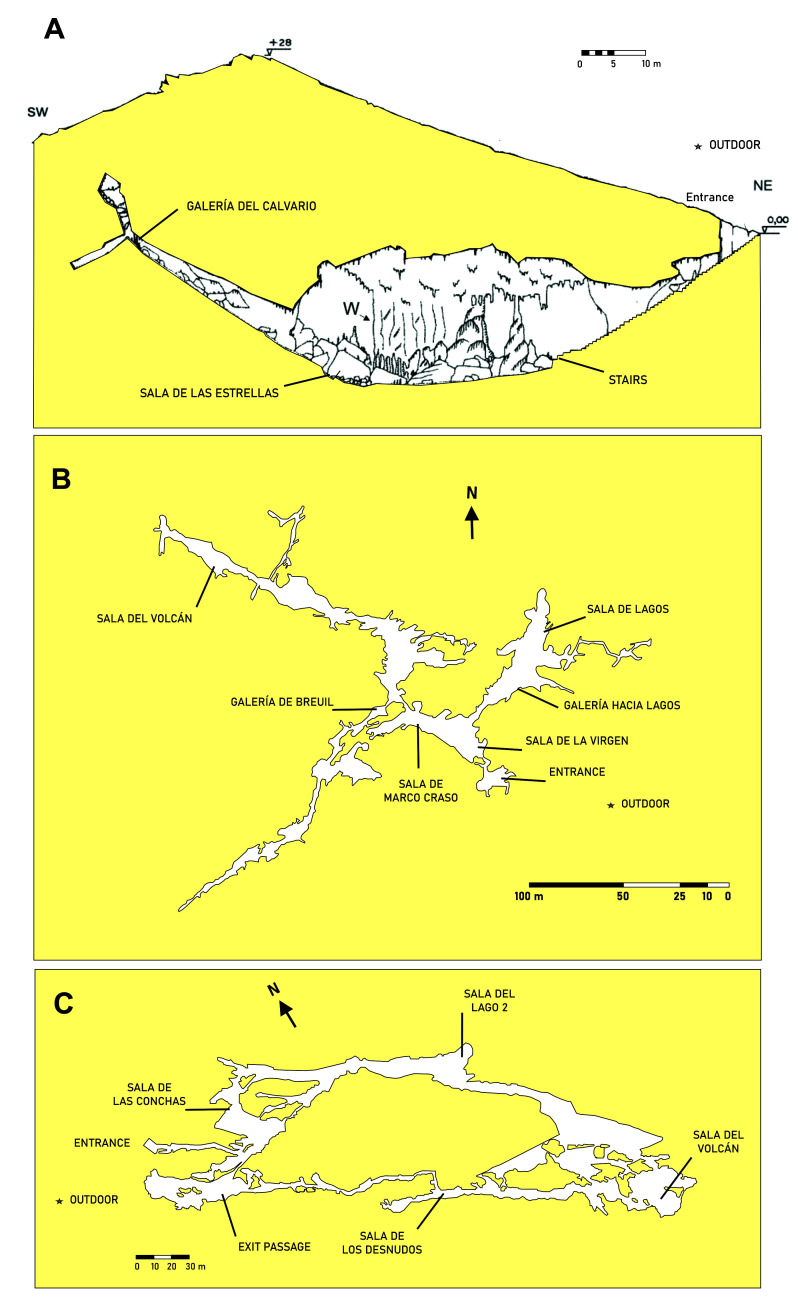
FIGURE 1: Map and sampling locations in the caves. **(A)***Cueva de Ardales*; **(B)***Cueva del Tesoro*; **(C)***Gruta de las Maravillas*.

In this paper we present an aerobiological study of three Andalusian show caves subjected to different managements. The caves cover examples from western to eastern Andalusia and are separated by more than 300 km. Samplings were carried out along four seasons and the colony-forming units per cubic meter (CFU m^-3^) in different galleries and rooms were assessed (**Figure 1**).

## RESULTS

**Figure 2:**
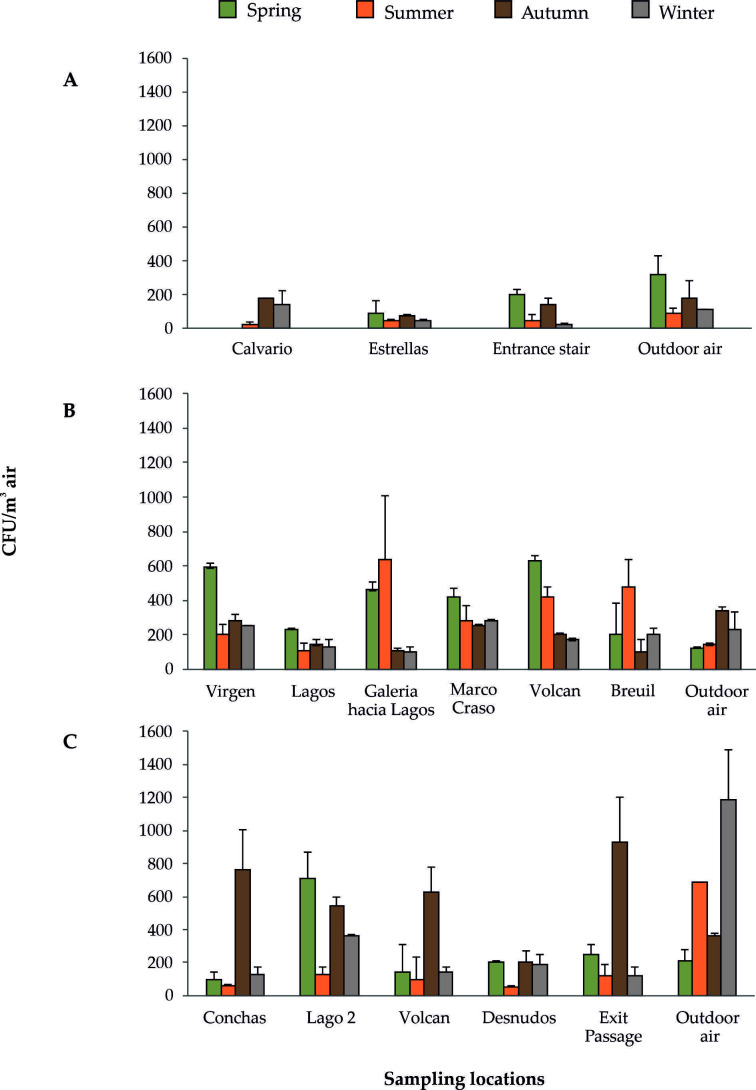
FIGURE 2: CFU m^−3^ of bacteria in the four seasonal samplings. **(A)***Cueva de Ardales*; **(B)***Cueva del Tesoro*; **(C)***Gruta de las Maravillas*.

There are large differences among the CFU m^-3^ in rooms and galleries, along the four seasons, in the three caves, as depicted in **Figure 2** and Supplementary Tables S1-S12. The cave less frequented by visitors (*Cueva de Ardales*) presented the lowest amount of bacteria in the air, with a maximum of 200 CFU m^-3^ counted at the end of the cave stair in spring, which was in agreement with the high quantity of bacteria outdoor (320 CFU m^-3^). Summer was the season with a lower amount of bacteria inside and outside the cave. Autumn and winter were the seasons with high and comparable airborne bacteria, both deepest in the cave and outside (180-110 CFU m^-3^), as corresponded to a ventilation period. In this period, the air outside is colder and denser with respect to inside and enters into the cave transporting airborne bacteria.

41 different species were recorded inside this cave and 40 outside. Only eight of the bacteria recorded outside were also found inside the cave.

The most abundant bacteria identified inside *Cueva de Ardales* were *Pseudarthrobacter* spp. and *Micrococcus luteus* in spring; *Bacillus altitudinis, Peribacillus simplex* and *Arthrobacter methylotrophus* in summer; *M. luteus* in autumn; *M. luteus* and *Arthrobacter citreus* in winter. Outdoor, the most abundant bacteria were *Pseudarthrobacter* spp. in spring, and summer, *Arthrobacter* spp. in winter, and *M. luteus* in autumn (Supplementary Tables S1-S4).

In *Cueva del Tesoro* the higher CFU m^-3^ of bacteria were obtained in the air of *Sala de la Virgen* and *Sala del Volcan* (spring) and *Galeria hacia Lagos* and *Galeria Breuil* (summer), whereas the lower one was recorded outdoor. In spring and summer, the cave suffered a stagnation period, which explains the high rates of bacteria inside. This stagnation period is produced when the external temperature becomes higher than the cave temperature and the convective air circulation stops. In autumn and winter, the higher CFU m^-3^ were obtained in *Sala de la Virgen* and *Sala de Marco Craso*, both next to the entrance, in agreement with the high number of bacteria outdoor. In these seasons, the entry of external cold air, typical of the ventilation period, has an enormous influence on the dispersal of outdoor airborne bacteria and their increase in the cave air. These stagnation and ventilation periods are common in caves.

In *Cueva del Tesoro* 130 different bacterial species were isolated from cave air and 34 outside, from which 17 were also recorded inside.

*M. luteus* was abundant in all the seasons, and *Micrococcus endophyticus* in spring and summer, but some other bacteria attained important abundances, such as *Knoellia locipacati* in spring and summer, and *Streptomyces kurssanovii* in autumn and winter. *M. luteus* was also abundant outdoor in spring, autumn and winter, whereas in summer *Brevibacterium aurantiacum, Glutamicibacter* spp. and *Pseudarthrobacter psychrotolerans* comprised about 63% of the abundance (Supplementary Tables S5-S8).

*Gruta de las Maravillas* exhibited a high abundance of bacteria in a few rooms. The greater CFU m^-3^ were found in autumn (*Sala de las Conchas* and *Exit Passage*), although other rooms also displayed important CFU m^-3^ (*Sala del Volcan, Sala del Lago 2*). In most rooms, the CFU m^-3^ was twice the number of bacteria outdoor. In winter, the abundance of airborne bacteria inside decreased with respect to autumn between 7.5 and 1.5 times, but outdoor the bacteria increased 3 times. In the stagnation period (spring and summer) the amount of bacteria in the air varied considerably. The lower abundances were obtained in summer; the maximum reached 130 CFU m^-3^ in *Sala del Lago 2*, while outside the amount was 690 CFU m^-3^. Slightly higher rates were recorded in spring (100-250 CFU m^-3^), except for *Sala del Lago 2*, with 710 CFU m^-3^, although outdoor the rate was the lowest (210 CFU m^-3^) of all the seasonal samplings. 94 species of bacteria were retrieved inside *Gruta de las Maravillas* and 48 outside, but only 18 of these were also isolated inside the cave.

Similar to *Cueva del Tesoro, M. luteus* and *Micrococcus endophyticus* were also the most abundant bacteria in *Gruta de las Maravillas*. Other relevant bacteria, including members of ten genera (*Aerococcus, Bacillus, Brevundimonas, Staphylococcus, Kocuria, Corynebacterium, Empedobacter, Microbacterium, Agrococcus, Glutamicibacter*) reached abundances above 20% in different rooms and seasons. Outside the cave a high diversity of bacteria was recorded of which *M. luteus* stood out (Supplementary Tables S9-S12).

## DISCUSSION

The wide difference in bacterial diversity inside and outside the three caves supports the assumption that the air is mostly populated by bacteria specific to the subterranean environment. In fact, *Cueva de Ardales* and *Gruta de las Maravillas* showed a coincidence of 19.5 and 19.1%, respectively, between inside and outside bacteria. *Cueva del Tesoro* only reached 12.6%.

The diversity of bacterial species in the three caves seems to be related with the presence of abundant phototrophic biofilms. Thus, *Cueva del Tesoro* exhibited three times the bacterial species as *Cueva de Ardales* and represented about one and half of those found in *Gruta de las Maravillas*, which is in accordance with the extent of biofilms in each cave.

The high abundance of bacteria in *Cueva del Tesoro* can be explained by two facts: i) the narrow galleries and halls which prevent a dilution effect of bacteria in the air, and ii) the high density of phototrophic biofilms, all over the cave. On the contrary, *Gruta de las Maravillas* holds much bigger rooms with considerable volume (i.e. rooms with a height greater than 40 m), and scarce phototrophic biofilms, which explains the reduced number of bacteria in the air. The low CFU m^-3^ of bacteria in *Cueva de Ardales* is consistent with the lack of phototrophic biofilms (no permanent lighting installation, and visited with pocket lamps), the high volume of the main hall (as seen in **Figure 1A**), and the low number of visitors.

The high amount of bacteria in *Sala del Lago 2* in spring (710 CFU m^-3^) could be explained by the thermal zoning of the interior of *Gruta de las Maravillas*, which responds to various factors: morphology of the cave that influences the ventilation capacity, distance from the entrance (**Figure 1C**), influence of visitors, and the existence of a big lake and a few other small lakes. In fact, the lowest temperatures in the cave were recorded in *Sala del Lago*, with values between 15.7°C and 16.1°C, which was attributed to a thermoregulatory effect of the lake's water mass [19]. This could stop the convective air circulation in *Sala del Lago*.

The high bacterial diversity was not associated with the number of visitors received by each cave, which was almost 5 times higher in *Gruta de las Maravillas* than in *Cueva del Tesoro*. However, *Cueva de Ardales* was the cave with a lesser amount of bacterial species and visitors, in addition that no phototrophic biofilms were observed; these factors might explain the lower diversity.

The genus *Micrococcus* is the most characteristic and abundant in the three show caves. For instance, in *Gruta de las Maravillas* (*Sala del Lago 2*) the bacterium reached up to 92% of abundance in summer, in *Cueva de Ardales* the abundance was over 50% in all rooms in autumn, and *Cueva del Tesoro* presented similar quantities in four out of six sampling points in spring (Supplementary Tables S1-S12).

This genus is commonly retrieved in European caves and the two most frequent species identified were *Micrococcus yunnanensis* and *M. luteus* [7, 20–23]. However, Huang *et al.* [24] proposed *M. yunnanensis* as later heterotypic synonym of *M. luteus*, based on the study of the whole-genome sequencing. In fact, both species are phenotypically and genotypically closely related and were differentiated by DNA-DNA hybridization, although for operative purposes were distinguished by their 16S rRNA sequences and the oxidase test, negative for *M. yunnanensis* and positive for *M. luteus*. Huang *et al.* [24], in addition to the reclassification as a single species, further considered *M. luteus* as oxidase-variable.

The wide distribution of *M. yunnanensis* in caves relies on the ability to grow at temperature as low as 4 °C (with an optimal growth temperature of 28°C), while *M. luteus* requires higher temperatures and the optimal growth temperature is 37°C. In their reclassification of *Micrococcus* species, Huang *et al.* [24] extended the temperature range of *M. luteus* from 4 to 45°C.

*M. yunnanensis* was retrieved from European caves [7, 22, 25] and *M. luteus* was recorded in European, Asian and African caves [26–28].

After *M. luteus, M. endophyticus* occupies the second rank in abundance in the three caves. This species was isolated from plant roots [29] and, as far as we know, was not previously reported in caves. Their presence in the air of three different caves supports an adaptation to subterranean niches, as suggested by the high CFU in the deepest galleries and their practical absence outside the cave.

Other less frequent *Micrococci* were *Micrococcus terreus* (present in the three caves) and *Micrococcus antarcticus* (in *Cueva del Tesoro* and *Gruta de las Maravillas*). Whereas *M. terreus* was isolated from different caves [13, 22], *M. antarcticus* was previously found in a coastal cave [13]. The optimum temperature for growth of this bacterium was 16.8 °C, which agrees with the temperatures of the caves [17].

Many species of the genus *Arthrobacter* were recently transferred to other genera: *Pseudarthrobacter, Glutamicibacter* or *Paenarthrobacter* [30]. The genera *Pseudarthrobacter, Glutamicibacter,* and *Arthrobacter* were the second most abundant in the caves. Contrary to the low number of *Micrococcus* species in the caves, for these four genera 28 different species were recorded from which nine species were not retrieved inside (Supplementary Tables S1-S12). Seven species (*P. phenanthrenivorans, Pseudarthrobacter oxydans, A. methylotrophus, A. citreus, Glutamicibacter mysorens, Glutamicibacter nicotianae* and *Glutamicibacter arilaitensis*) were found with abundances over 20% in the three caves. A few species were previously recorded in caves, such as *A. methylotrophus*, isolated from the air in the stair of *Cueva de Ardales* [11], and from the same place in this study, or *Glutamicibacter arilaitensis* in Lascaux Cave [7].

Furthermore, sequences with 98% similarity for *P. oxydans* were retrieved from an oligotrophic cave [31]. However, as far as we known, other *Arthrobacter* were not isolated from caves but from contaminated soils [32, 33].

The third most frequent genus was *Bacillus*, although the first in terms of biodiversity. 21 species of this genus were retrieved, 16 inside the cave and five more outside, but only four (*B. altitudinis, Bacillus idriensis, Bacillus aryabhattai* and *Bacillus vietnamensis*) attained abundances above 20% in any of the caves. Of interest was the identification of a new species: *Bacillus onubensis*. Two strains isolated from the air of *Cueva del Tesoro* and *Gruta de las Maravillas* were previously used for the description of this species [34]. Another new species, *Paracoccus cavernae*, was isolated from *Cueva de Ardales* [35].

*Bacillus* is one of the most abundant genus in caves all over the world [10, 11, 14, 36, 37]. Lavoie and Northup [38] correlated the presence of *Bacillus* in caves with high visitation levels, and Jurado *et al.* [39] associated this genus with the abundance of phototrophic biofilms. This is in aggreement with the presence of eleven different species in *Cueva del Tesoro*, seven in *Gruta de las Maravillas* and three in *Cueva de Ardales*, showing a direct relationship with the abundance of biofilms in each cave.

In addition, twelve bacterial genera related with the genus *Bacillus* were isolated from the caves: *Brevibacillus, Cytobacillus, Mesobacillus, Metabacillus, Neobacillus, Lysinibacillus, Paenibacillus, Peribacillus, Psychrobacillus, Solibacillus, Ureibacillus* and *Terribacillus*, although most of the members of these genera were recovered with abundances below 5%, except *Peribacillus simplex* (50%), *Paenibacillus amylolyticus* (10-12%), and *Lysinibacillus odyssey* (9.8%) in *Cueva del Tesoro*, and *Terribacillus goriensis* (8%) in *Gruta de las Maravillas* (Supplementary Tables S1-S12).

*Streptomyces* is one of the most abundant genus in caves, irrespective of the geographical situation [19, 40–44]. 22 species were recorded in this study, from which three (*Streptomyces lateritius, Streptomyces daghestanicus, Streptomyces spinoverrucosus*) were not found inside the caves. The number of species retrieved from the three caves showed a correlation with the abundance of biofilms as well (eleven in *Cueva del Tesoro*, five in *Gruta de las Maravillas* and three in *Cueva de Ardales*). Apart from *S. kurssanovii* that reached abundances as high as 60%, only five more species (*Streptomyces exfoliatus, Streptomyces anulatus, Streptomyces rochei, Streptomyces longisporoflavus, Streptomyces xantholiticus*) slightly surpassed 10% in *Cueva del Tesoro*; the remaining were well below 10% in all the three caves. Most of these six *Streptomyces* were found in other caves [21, 45–48].

Other abundant bacteria (over 20%) in the studied caves were *Terrabacter terrigena, Variovorax boronicumulans, Phyllobacterium ifriqiyense, Lentzea albidocapillata* subsp*. albidocapillata, K. locipacati, Knoellia subterranea, Jeotgalicoccus halophilus, Serratia liquefaciens, Sporosarcina luteola, Microbacterium esteraromaticum, Bhargavaea cecembensis, Aerococcus urinaeequi, Brevundimonas vesicularis*/*B. nasdae, Staphylococcus haemolyticus, Kocuria palustris, Staphylococcus saprophyticus* subsp*. bovis, Corynebacterium glutamicum, Empedobacter brevis, Microbacterium lacus,* and *Agrococcus baldri.* Most of these genera were reported in caves, but not all the species found in the three caves were previously recorded in similar environments [7, 13, 21, 49–52]. Interestingly, a few of these genera provided new species isolated from subterranean niches [53, 54], particularly *K. subterranea*, which reached 73.2% in *Cueva del Tesoro*. This bacterium was isolated and described from a Chinese cave [55]. The data indicate that caves are reservoirs for novel species of bacteria [34, 35, 54–56] as shown by the isolation of a novel *Paracoccus onubensis* from the walls of *Gruta de las Maravillas* [57].

To summarize, phototrophic biofilms seem to be the cause of the elevated abundance and diversity of bacteria in the caves and, at the same time, represent a danger for the conservation of the Paleolithic art and speleothems.

## CONCLUSION

In cultural heritage studies, a preventive conservation protocol includes the need to achieve aerobiological analysis for assessing the biological risk and to propose strategies aimed at controlling biodeterioration.

Aerobiology has proven to be a suitable approach for studying the dispersion of airborne bacteria in caves and to forecast the risks of eventual colonization of speleothems, walls and, subsequently, the biodeterioration of rock art.

The aerobiological study of three Andalusian caves shows large differences in CFU m^-3^ in rooms and galleries, along the four seasons of the year. These differences were related to the presence or absence of lighting and phototrophic biofilms and conditioned the diversity of bacteria in the caves. The diversity seems to be related with the abundant phototrophic biofilms, but not clearly associated with the number of visitors. The data show that the presence of phototrophic biofilms enhances the amount of bacteria in the air and the threat for the paintings and engravings. In addition, bacterial diversity data, inside and outside the three caves support that the cave air is mostly populated by species characteristic for the subterranean environment.

The data clearly indicate that phototrophic biofilms have to be removed, and the speleothems and walls cleaned. This should be accompanied by the use of a friendly illumination system to avoid the growth of cyanobacteria and algae, in order to protect the rock art.

## MATERIALS AND METHODS

Four aerobiological sampling campaigns were carried out between May 2011 and March 2012. In each cave campaign a representative number of galleries and/or rooms and an outdoor sampling point as control were selected, as indicated in **Figure 1** and Supplementary Tables S1-S12.

The methodology used was exhaustively described [4, 7]. A Surface Air System (Duo SAS, model Super 360) was used in the sampling [7]. Samples were taken in duplicates, at each sampling point and the volume of filtered air was set to 100 liters. This volume was selected because at higher volumes, colony-forming units per cubic meter (CFU m^-3^) were too high for counting [9]. The culture medium was trypticase-soy-agar (TSA, BD) with addition of cycloheximide (50 μg/mL; Applichem, Darmstad, Germany).

Counting and isolation of bacteria were accomplished for each colony type, using as selection criteria morphological characters. The bacteria were isolated in TSA culture medium.

The amounts of bacteria in each air sample were expressed as CFU m^-3^. The number of colonies counted was corrected for the statistical possibility of multiple particles passing through the same hole, following manufacturer's instructions.

Bacterial DNA was extracted and PCR products were sent to Macrogen Inc. (Amsterdam, The Netherlands) for sequencing. Molecular protocols and data were previously reported [7]. Briefly, DNA was extracted by dispersing a bacterial colony in 100 µl of 10mM TNE buffer, and freeze-thawing at -80°C and 65°C, respectively. For 16S rRNA amplification, the primers 616F (5'-AGAGTTTGATYMTGGCTCAG-3') [58] and 1510R (5'-GGTTACCTTGTTACGACTT-3' [59] were used. PCR amplifications were performed in a BioRad iCycler thermal cycler (BioRad, Hercules, CA, USA) using the following cycling parameters: 2 min of initial denaturing step at 95°C, followed by 35 cycles of denaturing (95°C for 1 min), annealing (55°C for 1 min) and extension (72°C for 2 min), with an additional extension step at 72°C for 10 min at the end.

For phylogenetic identification, the sequences were compared, using BLASTn algorithm, to the non-redundant databases of sequences deposited at the National Center for Biotechnology Information (NCBI). The sequences were deposited into the GenBank database under accession numbers MZ338588-MZ339147, LN650666, LN650668 and LN774332.

## AUTHOR CONTRIBUTION

Conceptualization, C.S.-J.; investigation, I.D-M; V.J.; M.A.R-C.; B.H.; writing—original draft preparation, C.S.-J.; writing—review and editing, C.S.-J.; All authors reviewed the results and approved the final version of the manuscript.

## SUPPLEMENTAL MATERIAL

Click here for supplemental data file.

All supplemental data for this article are available online at www.microbialcell.com/researcharticles/2021a-dominguez-monino-microbial-cell/.
