# Supplementary material for: Airborne bacteria in show caves from Southern Spain
Source: Microb Cell. 2021 Jul 26;8(10):247–55. doi: 10.15698/mic2021.10.762 (PMC8485469; doi:10.15698/mic2021.10.762)
Supplement: Supplementary file 1 [file mic-08-247-s01.pdf]

Supplementary Table S1. Sampling campaign in *Cueva de Ardales*, spring 2011. CFU/m^3, accession numbers, identifications and abundances.

Sampling	CFU/m^3 air	Accession Number	Identification (% identity)	Abundance (%)
Sala del Calvario	0			
Sala de las Estrellas	90±70	MZ338588	*Arthrobacter ruber* (JX949648; 99.5%)	5.6
		MZ338589	*Micrococcus luteus* (CP001628; 98.7%)	11.0
		MZ338590	*Micrococcus terreus* (jgi.1058018; 99.3%)	11.0
		LN650666	*Paracoccus cavernae* sp. nov. (LN650666; 100%)	5.6
		MZ338591	*Paracoccus marcusii* (Y12703; 99.9%)	5.6
		MZ338592	*Prolinoborus fasciculus* (JN175353; 99.9%)	5.6
		MZ338593	*Pseudarthrobacter phenanthrenivorans* (CP002379; 99.6%)	50.0
		MZ338594	*Streptomyces narbonensis* (AB184157; 99.4%)	5.6
End of stair	200±30	MZ338595	*Arthrobacter ruber* (JX949648; 98.8%)	2.6
		MZ338596	*Bacillus mycoides* (ACMU01000002; 99.3%)	2.6
		MZ338597	*Kocuria palustris* (Y16263; 100%)	5.3
		MZ338598	*Micrococcus luteus* (CP001628; 99.4%)	23.7
		MZ338599	*Paenibacillus glacialis* (EU815300; 98.7%)	2.6
		MZ338600	*Pseudarthrobacter oxydans* (X83408; 99.2%)	31.6
		MZ338601	*Pseudomonas zhaodongensis* (RFFM01000015; 98.7%)	2.6
		MZ338602	*Psychrobacillus psychrodurans* (jgi.1085849; 99.7%)	5.3
		MZ338603	*Rathayibacter festucae* (CP028137; 99.6%)	5.3
		MZ338604	*Staphylococcus cohnii* subsp. *cohnii* (D83361; 99.9%)	2.6
		MZ338605	*Staphylococcus hominis* subsp. *novobiosepticus* (AB233326; 99.9%)	13.2
		MZ338606	*Streptomyces pratensis* (JQ806215; 99.7%)	2.6
Outdoor air	320±110	MZ338607	*Arthrobacter bussei* (MN080869; 99.9%)	5.0
		MZ338608	*Arthrobacter humicola* (AB279890; 100%)	20.0
		MZ338609	*Bacillus mycoides* (ACMU01000002; 99.3%)	1.7
		MZ338610	*Clavibacter michiganensis* subsp. *chilensis* (KF663872)/ *C. michiganensis* subsp. *californiensis* (KF663871); (99.8%)	13.3
		MZ338611	*Curtobacterium flaccumfaciens* (AJ31209; 99.9%)	3.3
		MZ338612	*Frigoribacterium faeni* (BJUV01000064; 99.4%)	1.7
		MZ338613	*Paenarthrobacter aurescens* (BJMD01000050; 99.4%)	1.7
		MZ338614	*Paenibacillus stellifer* (CP009286; 97.7%)	1.7
		MZ338615	*Peribacillus simplex* (BCVO01000086; 99.5%)	8.3

Supplementary Table S1. Continuation

Sampling	CFU/m³ air	Accession Number	Identification (% identity)	Abundance (%)
Outdoor air	320±110	MZ338616	*Pseudarthrobacter oxydans* (X83408; 98.7%)	23.3
		MZ338617	*Pseudomonas kunmingensis* (JQ246444; 99.6%)	1.7
		MZ338618	*Rathayibacter festucae* (CP028137; 99.0%)	5.0
		MZ338619	*Solibacillus kalamii* (KT763359; 99.2%)	1.7
		MZ338620	*Staphylococcus cohnii* subsp. *urealyticus* (AB009936; 99.9%)	6.7
		MZ338621	*Terribacillus saccharophilus* (AB243845; 100%)	5.0

Supplementary Table S2. Sampling campaign in *Cueva de Ardales*, summer 2011. CFU/m^3, accession numbers, identifications and abundances.

Sampling	CFU/m^3 air	Accession Number	Identification (% identity)	Abundance (%)
Sala del Calvario	20±14	MZ338622	*Micrococcus endophyticus* (EU005372; 99.5%)	25.0
		MZ338623	*Peribacillus simplex* (BCVO01000086; 99.5%)	50.0
		MZ338624	*Terrabacter terrigena* (FJ423552; 97.7%)	25.0
Sala de las Estrellas	40±10	MZ338625	*Bacillus altitudinis* (ASJC01000029; 100%)	62.5
		MZ338626	*Ensifer numidicus* (AY500254; 99.7%)	12.5
		MZ338627	*Noviherbaspirillum canariense* (HQ830496; 98.7%)	12.5
		MZ338628	*Peribacillus simplex* (BCVO01000086; 100%)	12.5
End of stair	40±40	MZ338629	*Arthrobacter methylotrophus* (AF235090; 98.4%)	42.8
		MZ338630	*Metabacillus litoralis* (AY608605; 98.8%)	14.3
		MZ338631	*Prolinoborus fasciculus* (JN175353; 99.1%)	14.3
		MZ338632	*Variovorax boronicumulans* (AB300597; 99.9%)	28.6
Outdoor air	90±30	MZ338633	*Arthrobacter gyeryongensis* (JX141781; 99.1%)	16.6
		MZ338634	*Arthrobacter ruber* (JX949648; 99.7%)	5.6
		MZ338635	*Frigoribacterium faeni* (BJUV01000064; 98.6%)	5.6
		MZ338636	*Mesobacillus subterraneus* (RSFW01000004; 98.8%)	5.6
		MZ338637	*Micrococcus luteus* (CP001628; 99.8%)	5.6
		MZ338638	*Nocardia salmonicida* subsp. *cummidelens* (AF277202; 99.1%)	16.6
		MZ338639	*Peribacillus simplex* (BCVO01000086; 99.7%)	16.6
		MZ338640	*Pseudarthrobacter siccitolerans* (CAQI01000001; 99.0%)	22.2
		MZ338641	*Solibacillus isronensis* (AMCK01000046; 99.5%)	5.6

Supplementary Table S3. Sampling campaign in *Cueva de Ardales*, autumn 2011. CFU/m³, accession numbers, identifications and abundances.

Sampling	CFU/m³ air	Accession Number	Identification (% identity)	Abundance (%)
Sala del Calvario	180±0	MZ338642	*Arthrobacter methylotrophus* (AF235090; 98.7%)	3.0
		MZ338643	*Brevibacillus choshinensis* (LJJB01000010; 99.8%)	3.0
		MZ338644	*Macrococcus equipercicus* (Y15712; 99.6%)	3.0
		MZ338645	*Micrococcus luteus* (CP001628; 99.8%)	84.8
		MZ338646	*Streptomyces gardneri* (AB249908; 100%)	6.2
Sala de las Estrellas	70±10	MZ338647	*Aminobacter aminovorans* (AJ011759; 100%)	7.7
		MZ338648	*Brachybacterium paraconglomeratum* (AJ415377; 99.6%)	7.7
		MZ338649	*Micrococcus luteus* (CP001628; 99.8%)	53.8
		MZ338650	*Phyllobacterium ifriqiyense* (AY785325; 99.8%)	30.8
End of stair	140±40	MZ338651	*Bacillus proteolyticus* (MACH01000033; 99.9%)	11.1
		MZ338652	*Enterococcus faecium* (AJ301830; 99.2%)	7.4
		MZ338653	*Micrococcus luteus* (CP001628; 99.8%)	59.3
		MZ338654	*Pseudarthrobacter psychrotolerans* (MN559964; 99.9%)	11.1
		MZ338655	*Pseudomonas zhaodongensis* (RFFM01000015; 98.8%)	11.1
Outdoor air	180±100	MZ338656	*Agromyces cerinus* subsp. *cerinus* (jgi.1107671; 99.0%)	2.9
		MZ338657	*Bhargavaea cecembensis* (AM286423; 99.7%)	5.9
		MZ338658	*Brachybacterium ginsengisoli* (CP023564; 98.3%)	2.9
		MZ338659	*Brevundimonas vesicularis* (BCWM01000033)/ *B. nasdae* (AB071954); (99.6%)	2.9
		MZ338660	*Micrococcus luteus* (CP001628; 99.8%)	64.8
		MZ338661	*Pseudarthrobacter sulfonivorans* (AF235091; 99.4%)	5.9
		MZ338662	*Pseudomonas congelans* (FNJH01000022; 99.9%)	2.9
		MZ338663	*Pseudomonas lutea* (JRMB01000004; 99.7%)	2.9
		MZ338664	*Streptomyces exfoliatus* (AB184324; 99.9%)	8.9

Supplementary Table S4. Sampling campaign in *Cueva de Ardales*, winter 2012. CFU/m^3, accession numbers, identifications and abundances.

Sampling	CFU/m^3 air	Accession Number	Identification (% identity)	Abundance (%)
Sala del Calvario	140±85	MZ338665	*Acinetobacter lwoffii* (AIEL01000120; 100%)	7.1
		MZ338666	*Brevundimonas vesicularis* (BCWM01000033)/ *B. nasdae* (AB071954); (99.2%)	10.7
		MZ338667	*Micrococcus luteus* (CP001628; 99.7%)	57.1
		MZ338668	*Novosphingobium panipatense* (jgi.1118302; 99.6%)	10.7
		MZ338669	*Paracoccus marcusii* (Y12703; 99.8%)	14.4
Sala de las Estrellas	40±10	MZ338670	*Brevundimonas vesicularis* (BCWM01000033)/ *B. nasdae* (AB071954); (99.2%)	14.3
		MZ338671	*Micrococcus luteus* (CP001628; 99.7%)	57.1
		MZ338672	*Paracoccus marcusii* (Y12703; 100%)	14.3
		MZ338673	*Sporosarcina psychrophila* (D16277; 98.6%)	14.3
End of stair	20±10	MZ338674	*Arthrobacter citreus* (X80737; 100%)	66.7
		MZ338675	*Lentzea albidocapillata* subsp. *albidocapillata* (JOEA01000052; 99.7%)	33.3
Outdoor air	110±0	MZ338676	*Arthrobacter citreus* (X80737; 100%)	13.6
		MZ338677	*Arthrobacter globiformis* (BAEG01000072; 99.7%)	27.3
		MZ338678	*Kocuria rosea* (X87756; 99.7%)	13.6
		MZ338679	*Massilia aurea* (AM231588; 99.6%)	4.5
		MZ338680	*Novosphingobium panipatense* (jgi.1118302; 99.4%)	13.6
		MZ338681	*Paenibacillus purispatii* (EU888513; 98.7%)	4.5
		MZ338682	*Pseudomonas koreensis* (AF468452; 100%)	9.2
		MZ338683	*Rathayibacter tritici* (X77438; 99.5%)	9.2
		MZ338684	*Streptomyces spinoverrucosus* (AB184578; 99.6%)	4.5

Supplementary Table S5. Sampling campaign in *Cueva del Tesoro*, spring 2011. CFU/m^3, accession numbers, identifications and abundances.

Sampling	CFU/m^3 air	Accession Number	Identification (% identity)	Abundance (%)
Sala de La Virgen	590±28	MZ338685	*Falsirhodobacter halotolerans* (HE662814; 99.1%)	1.9
		MZ338686	*Microbacterium esteraromaticum* (Y17231; 98.4%)	1.9
		MZ338687	*Micrococcus luteus* (CP001628; 99.6%)	69.2
		MZ338688	*Pantoea eucalypti* (EF6880090; 99.7%)	2.9
		MZ338689	*Prolinoborus fasciculus* (JN175353; 99.9%)	12.5
		MZ338690	*Rhodococcus corynebacterioides* (AF430066; 98.6%)	1.9
		MZ338691	*Schumannella luteola* (AB362159; 98.1%)	1.9
		MZ338693	*Staphylococcus borealis* (MT586030; 99.5%)	3.8
		MZ338692	*Staphylococcus equorum* subsp. *equorum* (AB009939; 99.1%)	1.9
		MZ338694	*Staphylococcus saprophyticus* subsp. *bovis* (AB233327; 99.9%)	1.9
Sala de Lagos	230±10	MZ338695	*Bacillus idriensis* (AY904033; 99.3%)	4.7
		MZ338696	*Brachybacterium ginsengisoli* (CP023564; 99.5%)	4.7
		MZ338697	*Frigoribacterium faeni* (BJUV01000064; 99.4%)	7.0
		MZ338698	*Glutamicibacter halophytocola* (JX993762; 97.9%)	7.0
		MZ338699	*Microbacterium phyllosphaerae* (AJ277840; 99.5%)	16.3
		MZ338700	*Micrococcus endophyticus* (EU005372; 99.4%)	18.6
		MZ338701	*Paracoccus marcusii* (Y12703; 100%)	4.7
		MZ338702	*Prolinoborus fasciculus* (JN175353; 99.9%)	9.3
		MZ338703	*Pseudomonas cremoricolorata* (AB060137; 98.7%)	2.3
		MZ338704	*Psychrobacillus glaciei* (MN416951; 99.1%)	4.7
		MZ338705	*Rhodococcus corynebacterioides* (AF430066; 99.6%)	2.3
		MZ338706	*Staphylococcus epidermidis* (UHDF01000003; 99.7%)	4.7
		MZ338707	*Staphylococcus equorum* subsp. *equorum* (AB009939; 99.8%)	9.3
		MZ338708	*Williamsia deligens* (AJ920290; 98.8%)	4.7
Galería hacia Lagos	460±50	MZ338709	*Brevundimonas vesicularis* (BCWM01000033)/ *B. nasdae* (AB071954); (99.1%)	1.2
		MZ338710	*Glutamicibacter arilaitensis* (FQ311875; 99.7%)	15.7
		MZ338711	*Micrococcus luteus* (CP001628; 99.5%)	49.4
		MZ338712	*Prolinoborus fasciculus* (JN175353; 100%)	18.1
		MZ338713	*Psychrobacillus psychrodurans* (JGI.1085849; 99.7%)	1.2
		MZ338714	*Rhodococcus qingshengii* (LRRJ01000016; 99.6%)	8.4
		MZ338715	*Staphylococcus borealis* (MT586030; 99.5%)	1.2
		MZ338716	*Staphylococcus equorum* subsp. *equorum* (AB009939; 99.6%)	2.4
		MZ338717	*Zhihengliuella halotolerans* (DQ372937; 99.0%)	2.4

Supplementary Table S5. Continuation.

Sampling	CFU/m³ air	Accession Number	Identification (% identity)	Abundance (%)
Sala de Marco Craso	420±50	MZ338718	*Exiguobacterium mexicanum* (AM072764; 99.9%)	5.3
		MZ338719	*Glutamicibacter halophytocola* (JX993762; 99.3%)	2.7
		MZ338720	*Knoellia locipacati* (HQ171909; 98.2%)	25.3
		MZ338721	*Micrococcus luteus* (CP001628; 99.6%)	56.0
		MZ338722	*Prolinoborus fasciculus* (JN175353; 100%)	2.7
		MZ338723	*Staphylococcus equorum* subsp. *equorum* (AB009939; 99.9%)	6.7
		MZ338724	*Terribacillus goriensis* (DQ519571; 99.8%)	1.3
Sala del Volcán	630±30	MZ338725	*Exiguobacterium profundum* (AY818050; 100%)	2.7
		MZ338726	*Glutamicibacter arilaitensis* (FQ311875; 99.9%)	1.8
		MZ338727	*Massilia timonae* (AGZI01000009; 99.3%)	0.9
		MZ338728	*Micrococcus endophyticus* (EU005372; 99.2%)	49.1
		MZ338729	*Micrococcus luteus* (CP001628; 99.6%)	36.4
		MZ338730	*Prolinoborus fasciculus* (JN175353; 99.7%)	3.6
		MZ338731	*Pseudomonas atacamensis* (SSBS01000008; 99.2%)	1.8
		MZ338732	*Staphylococcus hominis* subsp. *novobiosepticus* (AB233326; 99.8%)	3.6
Galeria Breuil	200±180	MZ338733	*Knoellia locipacati* (HQ171909; 98.2%)	8.1
		MZ338734	*Metabacillus litoralis* (AY608605; 99.3%)	2.7
		MZ338735	*Microbacterium hydrocarbonoxydans* (BCRF01000095; 99.4%)	2.7
		MZ338736	*Microbacterium oxydans* (Y17227; 99.2%)	2.7
		MZ338737	*Micrococcus endophyticus* (EU005372; 99.3%)	35.1
		MZ338738	*Peribacillus loiseleuriae* (LFZW01000001; 98.5%)	10.8
		MZ338739	*Pseudarthrobacter psychrotolerans* (MN559964; 98.5%)	32.4
		MZ338740	*Rathayibacter festucae* (AM410683; 99.8%)	5.4
Outdoor air	120±10	MZ338741	*Bacillus taxi* (MK355518; 99.6%)	17.4
		MZ338742	*Citricoccus nitrophenolicus* (GU797177; 99.6%)	17.4
		MZ338743	*Luteimonas terrae* (KJ769177; 100%)	4.3
		MZ338744	*Metabacillus litoralis* (AY608605; 99.3%)	4.3
		MZ338745	*Micrococcus luteus* (CP001628; 99.9%)	34.8
		MZ338746	*Staphylococcus epidermidis* (UHDF01000003; 100%)	4.3
		MZ338747	*Staphylococcus hominis* subsp. *novobiosepticus* (AB233326; 99.9%)	13.0
		MZ338748	*Variovorax boronicumulans* (AB300597; 98.3%)	4.3

Supplementary Table S6. Sampling campaign in *Cueva del Tesoro*, summer 2011. CFU/m^3, accession numbers, identifications and abundances.

Sampling	CFU/m^3 air	Accession Number	Identification (% identity)	Abundance (%)
Sala de La Virgen	200±57	MZ338749	*Bacillus idriensis* (AY904033; 99.1%)	39.5
		MZ338750	*Cytobacillus oceanisediminis* (GQ292772; 99.4%)	2.6
		MZ338751	*Massilia timonae* (AGZI01000009; 99.3%)	2.6
		MZ338752	*Micrococcus luteus* (CP001628; 99.7%)	44.7
		MZ338753	*Streptomyces anulatus* (DQ026637)/ *S. fulvissimus* (CP005080)/ *S. microflavus* (AB184284)/ *S. cyaneofuscatus* (JOEM01000050)/ *S. halstedii* (AB184142)/ *S. fulvorobeus* (AB184711); (99.4%)	10.5
Sala de Lagos	110±40	MZ338754	*Knoellia locipacati* (HQ171909; 98.3%)	28.6
		MZ338755	*Massilia timonae* (U54470; 99.3%)	4.8
		MZ338756	*Micrococcus luteus* (CP001628; 99.5%)	38.1
		MZ338757	*Micrococcus terreus* (jgi.1058018; 99.8%)	4.8
		MZ338758	*Pseudomonas psychrotolerans* (FMWB01000061)/ *P. oryzihabitans* (BBIT01000012); (99.4%)	9.5
		MZ338759	*Zhihengliuella halotolerans* (DQ372937; 99.7%)	14.3
Galería hacia Lagos	640±370	LN774332	*Bacillus onubensis* (NSEB01000017; 99.7%)	2.7
		MZ338760	*Bacillus toyonensis* (CP006863)/ *B. mobilis* (MACF01000036); (99.9%)	0.9
		MZ338761	*Glutamicibacter arilaitensis* (FQ311875; 100%)	0.9
		MZ338762	*Knoellia subterranea* (AVPK01000001; 98%)	73.2
		MZ338763	*Micrococcus antarcticus* (AJ005932; 99.3%)	0.9
		MZ338764	*Micrococcus luteus* (CP001628; 99.6%)	8.9
		MZ338765	*Sphingomonas sanguinis* (BCTY01000091; 99.2%)	1.8
		MZ338766	*Staphylococcus caprae* (AB009935; 98.5%)	1.8
		MZ338767	*Staphylococcus cohnii* subsp. *urealyticus* (AB009936; 99.7%)	3.6
		MZ338768	*Streptomyces exfoliatus* (JNZP01000081; 99.7%)	5.4
Sala de Marco Craso	280±90	MZ338769	*Acinetobacter oryzae* (GU954428; 99.1%)	3.9
		MZ338770	*Brevundimonas vesicularis* (BCWM01000033)/ *B. nasdae* (AB071954); (99.0%)	2.0
		MZ338771	*Cytobacillus horneckiae* (FR749913; 100%)	2.0
		MZ338772	*Massilia suwonensis* (FJ969487; 98.8%)	7.8
		MZ338773	*Massilia timonae* (AGZI01000009; 99.1%)	2.0
		MZ338774	*Microbacterium phyllosphaerae* (AJ277840; 98.9%)	3.9
		MZ338775	*Micrococcus endophyticus* (EU005372; 99.3%)	66.7
		MZ338776	*Paracoccus aerius* (KX664462; 99.7%)	2.0
		MZ338777	*Pseudomonas kuykendallii* (FNNU01000014; 99.3%)	9.8

Supplementary Table S6. Continuation.

Sampling	CFU/m³ air	Accession Number	Identification (% identity)	Abundance (%)
Sala del Volcán	420±60	MZ338778	*Glutamicibacter arilaitensis* (FQ311875; 99.7%)	6.7
		MZ338779	*Massilia oculi* (FR773700; 99.4%)	4.0
		MZ338780	*Micrococcus endophyticus* (EU005372; 99.4%)	60.0
		MZ338781	*Staphylococcus cohnii* subsp. *cohnii* (D83361; 100%)	5.3
		MZ338782	*Staphylococcus cohnii* subsp. *urealyticus* (AB009936; 100%)	2.7
		MZ338783	*Staphylococcus hominis* subsp. *hominis* (X66101; 99.4%)	5.3
		MZ338784	*Staphylococcus saprophyticus* subsp. *bovis* (AB233327; 99.6%)	8.0
		MZ338785	*Streptomyces exfoliatus* (JNZP01000081; 99.2%)	2.7
		MZ338786	*Streptomyces virginiae* (JOAK01000082; 99.8%)	4.0
		MZ338787	*Ureibacillus chungkukjangi* (JX217747; 98.2%)	1.3
Galería Breuil	480±160	MZ338788	*Acinetobacter johnsonii* (APON01000005; 98.5%)	8.1
		MZ338789	*Bacillus licheniformis* (AE017333; 99.9%)	2.3
		MZ338790	*Glutamicibacter mysorens* (AJ639831; 99.7%)	44.2
		MZ338791	*Mesobacillus thioparans* (DQ371431; 98.8%)	3.5
		MZ338792	*Microbacterium aerolatum* (BJUW01000027; 99.3%)	4.7
		MZ338793	*Micrococcus luteus* (CP001628; 99.3%)	29.1
		MZ338794	*Peribacillus loiseleuriae* (LFZW01000001; 98.6%)	2.3
		MZ338795	*Pseudomonas xanthomarina* (AB176954; 99.0%)	5.8
Outdoor air	140±10	MZ338796	*Acinetobacter johnsonii* (APON01000005; 98.7%)	3.7
		MZ338797	*Brevibacterium aurantiacum* (X76566; 98.8%)	22.2
		MZ338798	*Glutamicibacter halophytocola* (JX993762; 99.2%)	3.7
		MZ338799	*Glutamicibacter mysorens* (AJ639831; 99.6%)	18.5
		MZ338800	*Metabacillus litoralis* (AY608605; 99.4%)	3.7
		MZ338801	*Microbacterium oleivorans* (BCRG01000019; 99.9%)	3.7
		MZ338802	*Micrococcus endophyticus* (EU005372; 99.4%)	3.7
		MZ338803	*Pseudarthrobacter psychrotolerans* (MN559964; 99.1%)	18.5
		MZ338804	*Sphingomonas yunnanensis* (AY894691; 98.9%)	3.7
		MZ338805	*Streptomyces exfoliatus* (JNZP01000081; 99.5%)	3.7
		MZ338806	*Zhihengliuella halotolerans* (DQ372937; 99.7%)	14.8

Supplementary Table S7. Sampling campaign in *Cueva del Tesoro*, autumn 2011. CFU/m^3, accession numbers, identifications and abundances.

Sampling	CFU/m^3 air	Accession Number	Identification (% identity)	Abundance (%)
Sala de La Virgen	280±35	MZ338807	*Bacillus proteolyticus* (MACH01000033; 98.0%)	2.0
		MZ338808	*Corynebacterium xerosis* (LAYS01000008; 99.9%)	3.9
		MZ338809	*Jeotgalicoccus halophilus* (FJ386517; 100%)	29.4
		MZ338810	*Kocuria rosea* (X87756; 99.2%)	9.8
		MZ338811	*Micrococcus luteus* (CP001628; 99.9%)	29.4
		MZ338812	*Novosphingobium panipatense* (jgi.1118302; 99.6%)	7.8
		MZ338813	*Paenisporosarcina quisquiliarum* (DQ333897; 98.8%)	7.8
		MZ338814	*Rhodococcus zopfii* (BCXI01000001; 98.7%)	2.0
		MZ338815	*Streptomyces prasinus* (LIRH01000162; 100%)	7.8
Sala de Lagos	140±30	MZ338816	*Arenivirga flava* (LC203064; 99.5%)	9.5
		MZ338817	*Arthrobacter pityocampae* (EU855749; 99.2%)	4.8
		MZ338818	*Brevundimonas vesicularis* (BCWM01000033)/ *B. nasdae* (AB071954); (99.6%)	4.8
		MZ338819	*Massilia niabensis* (EU808006; 98.9%)	4.8
		MZ338820	*Microbacterium phyllosphaerae* (AJ277840; 99.8%)	4.8
		MZ338821	*Micrococcus luteus* (CP001628; 99.8%)	9.5
		MZ338822	*Serratia liquefaciens* (CP006252; 99.9%)	33.3
		MZ338823	*Staphylococcus equorum* subsp. *equorum* (AB009939; 100%)	4.8
		MZ338824	*Staphylococcus xylosus* (MRZO01000018; 99.9%)	9.5
		MZ338825	*Streptomyces longisporoflavus* (AB184220; 99.9%)	14.3
Galería hacia Lagos	110±10	MZ338826	*Arthrobacter pokkalii* (KM507333; 99.2%)	23.8
		MZ338827	*Arthrobacter tumbae* (AJ315069; 100%)	14.3
		MZ338828	*Kocuria rosea* (X87756; 99.8%)	19.0
		MZ338829	*Microbacterium oleivorans* (BCRG01000019; 99.4%)	14.3
		MZ338830	*Sporosarcina luteola* (AB473560; 99.9%)	23.8
		MZ338831	*Streptomyces xantholiticus* (AB184349; 99.2%)	4.8
Sala de Marco Craso	250±10	MZ338833	*Bacillus tropicus* (MACG01000025; 99.8%)	14.6
		MZ338832	*Bacillus wiedmannii* (LOBC01000053)/ *B. albus* (MAOE01000087)/ *B. luti* (MACI01000041)/ *B. fungorum* (MG601116); (100%)	10.4
		MZ338834	*Glutamicibacter arilaitensis* (FQ311875; 100%)	12.5
		MZ338835	*Kocuria rosea* (X87756; 99.8%)	10.4
		MZ338836	*Microbacterium esteraromaticum* (Y17231; 99.8%)	20.8
		MZ338837	*Prolinoborus fasciculus* (JN175353; 99.9%)	8.3
		MZ338838	*Pseudoclavibacter helvolus* (X77440; 99.8%)	6.3
		MZ338839	*Streptomyces anulatus* (DQ026637; 99.7%)	6.3
		MZ338840	*Streptomyces exfoliatus* (AB184324; 99.9%)	10.4

Supplementary Table S7. Continuation.

Sampling	CFU/m³ air	Accession Number	Identification (% identity)	Abundance (%)
Sala del Volcán	200±10	MZ338841	*Glutamicibacter arilaitensis* (FQ311875; 99.7%)	7.9
		MZ338842	*Lentzea albidocapillata* subsp. *albidocapillata* (JOEA01000052; 99.2%)	2.6
		MZ338843	*Metabacillus litoralis* (AY608605; 99.3%)	7.9
		MZ338844	*Micrococcus luteus* (CP001628; 99.8%)	68.4
		MZ338845	*Paenisporosarcina quisquiliarum* (DQ333897; 99.8%)	2.6
		MZ338846	*Pseudarthrobacter oxydans* (X83408; 99.8%)	10.5
Galería Breuil	100±70	MZ338847	*Bacillus aryabhattai* (EF114313; 100%)	20.0
		MZ338848	*Micrococcus luteus* (CP001628; 99.8%)	20.0
		MZ338849	*Streptomyces kurssanovii* (AB184325; 99.9%)	60.0
Outdoor air	340±20	MZ338850	*Bacillus humi* (AJ627210; 99.0%)	6.3
		MZ338851	*Glutamicibacter arilaitensis* (FQ311875; 99.7%)	7.9
		MZ338852	*Microbacterium hydrocarbonoxydans* (BCRF01000095; 99.9%)	15.9
		MZ338853	*Micrococcus luteus* (CP001628; 99.8%)	41.3
		MZ338854	*Neobacillus bataviensis* (AJ542508)/ *N. soli* (BCVI01000121); (99.1%)	4.8
		MZ338855	*Pseudarthrobacter oxydans* (X83408; 99.7%)	3.2
		MZ338856	*Pseudomonas mediterranea* (AUPB01000004; 99.0%)	4.8
		MZ338857	*Sphingomonas aerolata* (AJ429240; 99.9%)	1.6
		MZ338858	*Streptomyces exfoliatus* (JNZP01000081; 99.5%)	9.5
		MZ338859	*Streptomyces lateritius* (AJ781326; 99.2%)	4.8

Supplementary Table S8. Sampling campaign in *Cueva del Tesoro*, winter 2012. CFU/m^3, accession numbers, identifications and abundances.

Sampling	CFU/m^3 air	Accession Number	Identification (% identity)	Abundance (%)
Sala de la Virgen	250±0	MZ338860	*Acinetobacter lwoffii* (AIEL01000120; 100%)	4.2
		MZ338861	*Arthrobacter agilis* (X80748; 99.8%)	2.1
		MZ338862	*Bacillus aryabhattai* (EF114313; 100%)	4.2
		MZ338863	*Bhargavaea cecembensis* (AM286423; 99.5%)	8.3
		MZ338864	*Kocuria polaris* (JSUH01000031; 99.7%)	4.2
		MZ338865	*Leuconostoc mesenteroides* subsp. *mesenteroides* (CP000414; 99.9%)	6.3
		MZ338866	*Lysinibacillus xylanilyticus* (FJ477040; 99.3%)	4.2
		MZ338867	*Micrococcus luteus* (CP001628; 99.8%)	22.9
		MZ338868	*Okibacterium fritillariae* (FUZP01000004; 99.9%)	16.7
		MZ338869	*Paenibacillus amylolyticus* (BIMJ01000009; 100%)	10.4
		MZ338870	*Pantoea dispersa* (DQ504305; 98.8%)	2.1
		MZ338871	*Sphingomonas olei* (KX672814; 100%)	4.2
		MZ338872	*Streptomyces cremeus* (AB184124; 98.9%)	6.3
		MZ338873	*Streptomyces koyangensis* (CP031742; 99.5%)	4.2
Sala de Lagos	130±40	MZ338874	*Frigoribacterium faeni* (BJUV01000064; 99.5%)	8.0
		MZ338875	*Glutamicibacter nicotianae* (BJNE01000038; 98.1 %)	32.0
		MZ338876	*Glutamicibacter soli* (EF660748; 98.9%)	16.0
		MZ338877	*Paenibacillus amylolyticus* (BIMJ01000009; 100%)	12.0
		MZ338878	*Planococcus ruber* (KX950835; 98.7%)	4.0
		MZ338879	*Pseudarthrobacter siccitolerans* (CAQI01000001; 100%)	16.0
		MZ338880	*Pseudomonas rhizosphaerae* (CP009533; 99.8%)	12.0
Galería hacia Lagos	100±30	MZ338881	*Arthrobacter crystallopoietes* (FNKH01000002; 99.2%)	15.0
		MZ338882	*Bhargavaea cecembensis* (AM286423; 99.5%)	20.0
		MZ338883	*Corynebacterium casei* (CP004350; 100%)	10.0
		MZ338884	*Kocuria gwangalliensis* (EU286964; 99.4%)	5.0
		MZ338885	*Kocuria polaris* (JSUH01000031; 99.7%)	10.0
		MZ338886	*Microbacterium paraoxydans* (BCRH01000180; 99.7%)	10.0
		MZ338887	*Micrococcus luteus* (CP001628; 99.8%)	15.0
		MZ338888	*Streptomyces rochei* (MUMD01000370)/ *S. vinaceusdrappus* (AY999929)/ *S. enissocaesilis* (DQ026641); (100%)	10.0
		MZ338889	*Streptomyces xantholiticus* (AB184349; 99.1%)	5.0

Supplementary Table S8. Continuation.

Sampling	CFU/m³ air	Accession Number	Identification (% identity)	Abundance (%)
Sala de Marco Craso	280±10	MZ338890	*Arthrobacter pascens* (X80740; 98.2%)	13.7
		MZ338891	*Bacillus cereus* (AE016877)/ *B. paramycoides* (MAOI01000012)/ *B. paranthracis* (MACE01000012)/ *B. nitratireducens* (KJ812430); (100%)	7.8
		MZ338892	*Bhargavaea cecembensis* (AM286423; 99.5%)	5.9
		MZ338893	*Kocuria gwangalliensis* (EU286964; 99.9%)	2.0
		MZ338894	*Lysinibacillus odysseyi* (AF526913; 98.7%)	9.8
		MZ338895	*Microbacterium paraoxydans* (BCRH01000180; 100%)	3.9
		MZ338896	*Micrococcus luteus* (CP001628; 99.8%)	15.7
		MZ338897	*Paracoccus marcusii* (Y12703)/ *P. carotinifaciens* (AB006899); (100%)	9.8
		MZ338898	*Planococcus ruber* (KX950835; 98.7%)	2.0
		MZ338899	*Psychrobacillus psychrodurans* (jgi.1085849; 97.6%)	3.9
		MZ338900	*Staphylococcus epidermidis* (UHDF01000003; 100%)	5.9
		MZ338901	*Staphylococcus equorum* subsp. *equorum* (AB009939; 100%)	2.0
		MZ338902	*Streptomyces prasinus* (LIRH01000162; 99.9%)	5.9
		MZ338903	*Streptomyces xantholiticus* (AB184349; 99.2%)	11.8
Sala del Volcán	170±10	MZ338904	*Alcaligenes faecalis* subsp. *phenolicus* (AUBT01000026; 99.7%)	6.3
		MZ338905	*Brevundimonas vesicularis* (BCWM01000033)/ *B. nasdae* (AB071954); (99.5%)	6.3
		MZ338906	*Metabacillus indicus* (JGVU01000003; 100%)	12.1
		MZ338907	*Metabacillus litoralis* (AY608605; 99.1%)	3.1
		MZ338908	*Micrococcus luteus* (CP001628; 99.8%)	40.6
		MZ338909	*Paenibacillus amylolyticus* (BIMJ01000009; 100%)	3.1
		MZ338910	*Streptomyces cinereoruber* subsp. *cinereoruber* (AB184121; 100%)	3.1
		MZ338911	*Streptomyces kurssanovii* (AB184325; 99.1%)	25.0
Galeria Breuil	200±40	MZ338912	*Agrobacterium cavarae* (MK940276; 99.8%)	5.9
		MZ338913	*Aureimonas altamirensis* (BBWQ01000019; 100%)	5.9
		MZ338914	*Bacillus foraminis* (AJ717382; 99.8%)	8.8
		MZ338915	*Bacillus halmapalus* (KV917375; 99.1%)	8.8
		MZ338916	*Brevundimonas vesicularis* (BCWM01000033)/ *B. nasdae* (AB071954); (99.5%)	5.9
		MZ338917	*Glutamicibacter arilaitensis* (FQ311875; 99.2%)	2.9
		MZ338918	*Massilia oculi* (FR773700; 100%)	2.9
		MZ338919	*Metabacillus litoralis* (AY608605; 99.2%)	2.9
		MZ338920	*Microbacterium yannicii* (FN547412; 99.0%)	2.9

Supplementary Table S8. Continuation.

Sampling	CFU/m³ air	Accession Number	Identification (% identity)	Abundance (%)
Galeria Breuil		MZ338921	*Micrococcus luteus* (CP001628; 99.8%)	26.5
		MZ338922	*Pseudarthrobacter oxydans* (X83408; 99.8%)	8.8
		MZ338923	*Pseudomonas congelans* (FNJH01000022; 98.4%)	11.8
		MZ338924	*Psychrobacillus insolitus* (AM980508; 98.8%)	2.9
		MZ338925	*Rhodococcus corynebacterioides* (AF430066; 99.6%)	2.9
Outdoor air	230±100	MZ338926	*Acinetobacter lwoffii* (AIEL01000120; 99.8%)	11.4
		MZ338927	*Bacillus badius* (JXLP01000009; 99.2%)	2.3
		MZ338928	*Bacillus cereus* (AE016877; 100%)	9.1
		MZ338929	*Cellulosimicrobium funkei* (AY501364; 99.8%)	6.8
		MZ338930	*Citricoccus nitrophenolicus* (GU797177; 98.7%)	6.8
		MZ338931	*Micrococcus luteus* (CP001628; 99.7%)	20.5
		MZ338932	*Peribacillus simplex* (AB363738; 99.8%)	9.1
		MZ338933	*Planococcus donghaensis* (CP016544; 98.4%)	2.3
		MZ338934	*Pseudarthrobacter scleromae* (AF330692; 98.9%)	15.9
		MZ338935	*Ureibacillus sinduriensis* (FJ169465; 99.6%)	15.9

Supplementary Table S9. Sampling campaign in *Gruta de las Maravillas*, spring 2011. CFU/m^3, accession numbers, identifications and abundances.

Sampling	CFU/m^3 air	Accession Number	Identification (% identity)	Abundance (%)
Sala de Las Conchas	100±42	MZ338936	*Acinetobacter lwoffii* (AIEL01000120; 100%)	10.0
		MZ338937	*Corynebacterium xerosis* (LAYS01000008; 99.4%)	15.0
		MZ338938	*Micrococcus luteus* (CP001628; 98.8%)	45.0
		MZ338939	*Pseudomonas asplenii* (LT629777; 99.1%)	5.0
		MZ338940	*Staphylococcus borealis* (MT586030; 99.9%)	15.0
		MZ338941	*Staphylococcus hominis* subsp. *novobiosepticus* (AB233326; 99.8%)	10.0
Sala del Lago 2	710±160	MZ338942	*Acinetobacter johnsonii* (APON01000005; 99.2%)	10.7
		MZ338943	*Aerococcus urinaeequi* (CP014162; 99.2%)	24.0
		MZ338944	*Kocuria tytonis* (MG547562; 99.6%)	9.1
		MZ338945	*Massilia suwonensis* (FJ969487; 99.1%)	0.8
		MZ338946	*Microbacterium foliorum* (JYIU01000006; 98.7%)	5.0
		MZ338947	*Micrococcus antarcticus* (AJ005932; 99.2%)	4.1
		MZ338948	*Micrococcus endophyticus* (EU005372; 99.1%)	35.5
		MZ338949	*Prolinoborus fasciculus* (JN175353; 99.6%)	2.5
		MZ338950	*Staphylococcus borealis* (MT586030; 99.2%)	8.3
Sala del Volcán	140±170	MZ338951	*Aerococcus urinaeequi* (CP014162; 99.6%)	7.1
		MZ338952	*Agrococcus baldri* (FOZN01000001; 98.2%)	3.6
		MZ338953	*Bacillus vietnamensis* (AB099708; 99.3%)	21.4
		MZ338954	*Corynebacterium casei* (CP004350; 99.7%)	3.6
		MZ338955	*Curtobacterium flaccumfaciens* (AJ312209; 99.8%)	10.7
		MZ338956	*Metabacillus litoralis* (AY608605; 98.8%)	3.6
		MZ338957	*Micrococcus endophyticus* (EU005372; 99.1%)	50.0
Sala de Los Desnudos	200±10	MZ338958	*Brevundimonas vesicularis* (BCWM01000033)/ *B. nasdae* (AB071954); (99.0%)	26.3
		MZ338959	*Micrococcus antarcticus* (AJ005932; 99.1%)	15.8
		MZ338960	*Micrococcus endophyticus* (EU005372; 99.1%)	15.8
		MZ338961	*Prolinoborus fasciculus* (JN175353; 99.9%)	13.2
		MZ338962	*Pseudomonas stutzeri* (CP002881; 99.9%)	5.3
		MZ338963	*Staphylococcus haemolyticus* (LILF01000056; 99.6%)	21.1
		MZ338964	*Staphylococcus saprophyticus* subsp. *bovis* (AB233327; 99.8%)	2.6

Supplementary Table S9. Continuation.

Sampling	CFU/m³ air	Accession Number	Identification (% identity)	Abundance (%)
Exit Passage	250±60	MZ338965	*Aerococcus urinaeequi* (CP014162; 100%)	6.3
		MZ338966	*Brevundimonas diminuta* (GL883089; 99.4%)	16.7
		MZ338967	*Micrococcus endophyticus* (EU005372; 99.2%)	58.3
		MZ338968	*Psychrobacter celer* (AY842259; 99.9%)	14.6
		MZ338969	*Rhodococcus fascians* (JMEN01000010; 99.5%)	2.1
		MZ338970	*Staphylococcus saprophyticus* subsp. *bovis* (AB233327; 99.9%)	2.1
Outdoor air	210±70	MZ338971	*Acinetobacter halotolerans* (KT032155; 98.5%)	21.6
		MZ338972	*Frigoribacterium faeni* (BJUV01000064; 99.9%)	21.6
		MZ338973	*Massilia timonae* (AGZI01000009; 99.5%)	2.7
		MZ338974	*Micrococcus endophyticus* (EU005372; 99.2%)	16.2
		MZ338975	*Micrococcus luteus* (CP001628; 99.9%)	24.3
		MZ338976	*Pseudomonas simiae* (AJ936933; 99.8%)	5.4
		MZ338977	*Staphylococcus edaphicus* (KY315825; 100%)	2.7
		MZ338978	*Staphylococcus equorum* subsp. *equorum* (AB009939; 99.3%)	5.4

Supplementary Table S10. Sampling campaign in *Gruta de las Maravillas*, summer 2011. CFU/m^3, accession numbers, identifications and abundances.

Sampling	CFU/m^3 air	Accession Number	Identification (% identity)	Abundance (%)
Sala de Las Conchas	60±7	MZ338979	*Kocuria palustris* (Y16263; 100%)	27.3
		MZ338980	*Micrococcus endophyticus* (EU005372; 99.2%)	36.4
		MZ338981	*Pseudarthrobacter siccitolerans* (CAQI01000001; 99.1%)	9.1
		MZ338982	*Staphylococcus hominis* subsp. *novobiosepticus* (AB233326; 99.3%)	18.2
		MZ338983	*Streptomyces spiroverticillatus* (AB249921; 99.0%)	9.1
Sala del Lago 2	130±40	MZ338984	*Micrococcus luteus* (CP001628; 99.7%)	92.0
		MZ338985	*Terribacillus goriensis* (DQ519571; 100%)	8.0
Sala del Volcán	100±130	MZ338986	*Aerococcus urinaeequi* (CP014162; 99.8%)	10.0
		MZ338987	*Bacillus mycoides* (ACMU01000002; 99.4%)	5.0
		MZ338988	*Microbacterium oleivorans* (BCRG01000019; 99.7%)	5.0
		MZ338989	*Micrococcus endophyticus* (EU005372; 99.4%)	50.0
		MZ338990	*Staphylococcus saprophyticus* subsp. *bovis* (AB233327; 99.4%)	30.0
Sala de Los Desnudos	50±10	MZ338991	*Corynebacterium glutamicum* (BA000036; 99.9%)	55.6
		MZ338992	*Micrococcus endophyticus* (EU005372; 99.5%)	44.4
Exit Passage	120±70	MZ338993	*Aerococcus urinaeequi* (CP014162; 99.9%)	20.8
		LN650668	*Bacillus onubensis* sp. nov. (NSEB01000017)	4.2
		MZ338994	*Empedobacter brevis* (AM177497; 99.5%)	20.8
		MZ338995	*Kaistella montana* (KX082815; 98.9%)	12.5
		MZ338996	*Knoellia subterranea* (AVPK01000001; 98.8%)	12.5
		MZ338997	*Micrococcus endophyticus* (EU005372; 99.5%)	20.8
		MZ338998	*Pseudomonas stutzeri* (CP002881; 99.7%)	8.3
Outdoor air	690±0	MZ338999	*Arthrobacter oryzae* (AB279889; 99.9%)	14.4
		LN650668	*Bacillus onubensis* sp. nov. (NSEB01000017)	5.9
		MZ339000	*Bacillus zhangzhouensis* (JOTP01000061; 99.7%)	3.4
		MZ339001	*Chryseomicrobium aureum* (KF781632; 99.4%)	10.2
		MZ339002	*Corynebacterium glutamicum* (BA000036; 99.9%)	2.5
		MZ339003	*Kocuria salina* (LT674162; 99.2%)	0.8
		MZ339004	*Massilia brevitalea* (EF546777; 98.8%)	4.2
		MZ339005	*Metabacillus litoralis* (AY608605; 99.2%)	0.8
		MZ339006	*Microbacterium esteraromaticum* (Y17231; 99.8%)	17.8
		MZ339007	*Microbacterium hydrocarbonoxydans* (BCRF01000095; 99.1%)	5.1

Supplementary Table S10. Continuation.

Sampling	CFU/m³ air	Accession Number	Identification (% identity)	Abundance (%)
Outdoor air		MZ339008	*Micrococcus endophyticus* (EU005372; 99.4%)	11.0
		MZ339009	*Paenibacillus lautus* (BIMF01000051; 99.6%)	5.9
		MZ339010	*Pseudomonas stutzeri* (CP002881; 99.6%)	2.5
		MZ339011	*Staphylococcus cohnii* subsp. *cohnii* (D83361; 99.9%)	14.4
		MZ339012	*Staphylococcus succinus* (AF004220; 99.7%)	0.8

Supplementary Table S11. Sampling campaign in *Gruta de las Maravillas*, autumn 2011. CFU/m^3, accession numbers, identifications and abundances.

Sampling	CFU/m^3 air	Accession Number	Identification (% identity)	Abundance (%)
Sala de Las Conchas	760±247	MZ339013	*Acinetobacter haemolyticus* (APQQ01000002; 99.4%)	0.8
		MZ339014	*Aerococcus urinaeequi* (CP014162; 99.8%)	2.4
		MZ339015	*Bacillus siamensis* (AJVF01000043; 99.9%)	0.8
		MZ339016	*Curtobacterium flaccumfaciens* (AJ312209; 100%)	5.5
		MZ339017	*Kocuria arsenatis* (KM874399; 99.9%)	2.4
		MZ339018	*Massilia timonae* (AGZI01000009; 99.4%)	3.9
		MZ339019	*Micrococcus endophyticus* (EU005372; 99.2%)	74.8
		MZ339020	*Pseudomonas caspiana* (LOHF01000033; 99.8%)	0.8
		MZ339021	*Psychrobacter faecalis* (AJ421528; 99.8%)	5.5
		MZ339022	*Staphylococcus hominis* subsp. *novobiosepticus* (AB233326; 99.9%)	1.6
		MZ339023	*Staphylococcus saprophyticus* subsp. *saprophyticus* (AP008934; 99.9%)	1.6
Sala del Lago 2	540±60	MZ339024	*Acinetobacter johnsonii* (APON01000005; 99.3%)	2.1
		MZ339025	*Aerococcus urinaeequi* (CP014162; 100%)	5.2
		MZ339026	*Haematobacter massiliensis* (DQ342309; 100%)	7.3
		MZ339027	*Kaistella montana* (KX082815; 98.9%)	7.3
		MZ339028	*Massilia suwonensis* (FJ969487; 99.3%)	6.3
		MZ339029	*Microbacterium aurum* (CP018762; 98.6%)	2.1
		MZ339030	*Micrococcus luteus* (CP001628; 99.6%)	63.5
		MZ339031	*Roseomonas mucosa* (UGVN01000001; 100%)	5.2
		MZ339032	*Streptomyces gardneri* (AB249908; 100%)	1.0
Sala del Volcán	630±150	MZ339033	*Acinetobacter lwoffii* (AIEL01000120; 99.9%)	3.7
		MZ339034	*Aerococcus urinaeequi* (CP014162; 100%)	7.3
		MZ339035	*Chryseobacterium tructae* (FR871429; 98.1%)	1.8
		MZ339036	*Luteimonas terrae* (KJ769177; 99.8%)	1.8
		MZ339037	*Massilia haematophila* (AM774589; 99.0%)	3.7
		MZ339038	*Massilia timonae* (AGZI01000009; 99.5%)	4.6
		MZ339039	*Microbacterium lacus* (AB286030; 99.3%)	25.7
		MZ339040	*Micrococcus luteus* (CP001628; 99.8%)	35.8
		MZ339042	*Rahnella victoriana* (KF308403; 99.8%)	11.0
		MZ339041	*Paracoccus caeni* (GQ250442; 100%)	4.6

Supplementary Table S11. Continuation.

Sampling	CFU/m³ air	Accession Number	Identification (% identity)	Abundance (%)
Sala de Los Desnudos	200±70	MZ339043	*Brachybacterium paraconglomeratum* (AJ415377; 99.5%)	2.6
		MZ339044	*Brevundimonas vesicularis* (BCWM01000033)/ *B. nasdae* (AB071954); (99.7%)	5.3
		MZ339045	*Massilia suwonensis* (FJ969487; 98.7%)	7.9
		MZ339046	*Microbacterium phyllosphaerae* (AJ277840; 98.9%)	2.6
		MZ339047	*Micrococcus luteus* (CP001628; 99.7%)	63.2
		MZ339048	*Paenibacillus uliginis* (FN556467; 99.4%)	2.6
		MZ339049	*Pseudomonas moorei* (AM293566; 97.9%)	5.3
		MZ339050	*Staphylococcus edaphicus* (KY315825; 99.6%)	10.5
Exit Passage	930±270	MZ339051	*Aerococcus urinaeequi* (CP014162; 99.9%)	7.2
		MZ339052	*Bacillus altitudinis* (ASJC01000029; 99.5%)	8.6
		MZ339053	*Corynebacterium xerosis* (LAYS01000008; 100%)	0.7
		MZ339054	*Kocuria rosea* (X87756; 98.7%)	4.6
		MZ339055	*Microbacterium esteraromaticum* (Y17231; 99.8%)	5.9
		MZ339056	*Micrococcus luteus* (CP001628; 99.8%)	63.8
		MZ339057	*Prolinoborus fasciculus* (JN175353; 99.9%)	6.6
		MZ339058	*Pseudomonas stutzeri* (CP002881; 99.9%)	0.7
		MZ339059	*Staphylococcus edaphicus* (KY315825; 100%)	2.0
Outdoor air	360±20	MZ339060	*Acinetobacter halotolerans* (KT032155; 98.7%)	9.6
		MZ339061	*Aerococcus urinaeequi* (CP014162; 99.9%)	6.5
		MZ339062	*Arthrobacter ulcerisalmonis* (MK211245; 98.9%)	4.8
		MZ339063	*Exiguobacterium mexicanum* (AM072764; 100%)	9.6
		MZ339064	*Micrococcus luteus* (CP001628; 99.8%)	50.0
		MZ339065	*Neobacillus niacini* (AB021194; 99.1%)	3.3
		MZ339066	*Rhodococcus corynebacterioides* (AF430066; 99.7%)	1.6
		MZ339067	*Sphingomonas olei* (KX672814; 99.6%)	6.5
		MZ339068	*Staphylococcus equorum* subsp. *equorum* (AB009939; 100%)	3.3
		MZ339069	*Staphylococcus saprophyticus* subsp. *saprophyticus* (AP008934; 99.8%)	4.8

Supplementary Table S12. Sampling campaign in *Gruta de las Maravillas*, winter 2012. CFU/m^3, accession numbers, identifications and abundances.

Sampling	CFU/m^3 air	Accession Number	Identification (% identity)	Abundance (%)
Sala de Las Conchas	130±42	MZ339070	*Acinetobacter lwoffii* (AIEL01000120; 99.9%)	3.8
		MZ339071	*Agrococcus baldri* (FOZN01000001; 99.4%)	23.1
		MZ339072	*Arthrobacter globiformis* (BAEG01000072; 99.5%)	3.8
		MZ339073	*Brevundimonas vesicularis* (BCWM01000033)/ *B. nasdae* (AB071954); (99.3%)	7.7
		MZ339074	*Macrococcus equipercicus* (Y15712; 100%)	3.8
		MZ339076	*Micrococcus luteus* (CP001628; 99.5%)	38.5
		MZ339075	*Micrococcus terreus* (jgi.1058018; 99.9%)	7.7
		MZ339077	*Staphylococcus equorum* subsp. *equorum* (AB009939; 99.8%)	7.7
		MZ339078	*Streptomyces avidinii* (AB184395; 99.8%)	3.8
Sala del Lago 2	360±10	MZ339079	*Acinetobacter baumannii* (ACQB01000091; 97.7%)	1.5
		MZ339080	*Acinetobacter johnsonii* (APON01000005; 99.4%)	4.6
		MZ339081	*Aerococcus urinaeequi* (CP014162; 100%)	10.8
		MZ339082	*Arthrobacter citreus* (X80737; 100%)	7.7
		MZ339083	*Bacillus licheniformis* (AE017333; 99.5%)	1.5
		MZ339084	*Bacillus megaterium* (JJMH01000057; 99.8%)	3.1
		MZ339085	*Brevundimonas vesicularis* (BCWM01000033)/ *B. nasdae* (AB071954); (99.3%)	1.5
		MZ339086	*Corynebacterium xerosis* (LAYS01000008; 99.9%)	1.5
		MZ339087	*Glutamicibacter arilaitensis* (FQ311875; 100%)	1.5
		MZ339088	*Janibacter limosus* (Y08539; 98.8%)	10.8
		MZ339089	*Kaistella anthropi* (AM982786; 98.1%)	4.6
		MZ339090	*Macrococcus equipercicus* (Y15712; 100%)	3.1
		MZ339091	*Microbacterium aurum* (CP018762; 98.6%)	7.7
		MZ339092	*Micrococcus luteus* (CP001628; 99.7%)	24.6
		MZ339093	*Planococcus rifietoensis* (CP013659; 100%)	3.1
		MZ339095	*Sporosarcina luteola* (AB473560; 100%)	4.6
		MZ339094	*Staphylococcus borealis* (MT586030; 99.9%)	7.7

Supplementary Table S12. Continuation.

Sampling	CFU/m³ air	Accession Number	Identification (% identity)	Abundance (%)
Sala del Volcán	140±30	MZ339096	*Alcaligenes faecalis* subsp. *phenolicus* (AUBT01000026; 99.2%)	4.0
		MZ339097	*Brachybacterium paraconglomeratum* (AJ415377; 99.9%)	12.0
		MZ339098	*Brevundimonas vesicularis* (BCWM01000033)/ *B. nasdae* (AB071954); (99.4%)	8.0
		MZ339099	*Glutamicibacter arilaitensis* (FQ311875; 100%)	28.0
		MZ339100	*Kaistella anthropi* (AM982786; 97.9%)	4.0
		MZ339101	*Microbacterium ginsengiterrae* (EU873314; 99.5%)	12.0
		MZ339102	*Micrococcus luteus* (CP001628; 99.7%)	12.0
		MZ339103	*Prolinoborus fasciculus* (JN175353; 98.6%)	8.0
		MZ339104	*Sphingobacterium lactis* (jgi.1085797; 99.8%)	4.0
		MZ339105	*Streptomyces xantholiticus* (AB184349; 99.2%)	8.0
Sala de Los Desnudos	190±60	MZ339106	*Achromobacter piechaudii* (BCTK01000022; 99.8%)	8.8
		MZ339107	*Acinetobacter halotolerans* (KT032155; 98.7%)	2.9
		MZ339108	*Acinetobacter ursingii* (AIEA01000080; 100%)	2.9
		MZ339109	*Brevundimonas vesicularis* (BCWM01000033)/ *B. nasdae* (AB071954); (99.7%)	17.7
		MZ339110	*Dietzia timorensis* (LMTB01000088; 99.9%)	2.9
		MZ339111	*Microbacterium aurum* (CP018762; 98.6%)	23.5
		MZ339112	*Micrococcus luteus* (CP001628; 99.7%)	32.4
		MZ339113	*Promicromonospora xylanilytica* (FJ214352; 99.6%)	2.9
		MZ339114	*Staphylococcus simulans* (D83373; 99.8%)	5.9
Exit Passage	120±50	MZ339115	*Brevundimonas vesicularis* (BCWM01000033)/ *B. nasdae* (AB071954); (99.6%)	8.7
		MZ339116	*Cupriavidus oxalaticus* (AF155567; 99.4%)	4.3
		MZ339117	*Glutamicibacter arilaitensis* (FQ311875; 100%)	4.3
		MZ339118	*Limimaricola cinnabarinus* (BATB01000114; 96.6%)	4.3
		MZ339119	*Macrococcus equipercicus* (Y15712; 99.9%)	4.3
		MZ339120	*Microbacterium aurum* (CP018762; 98.7%)	13.0
		MZ339121	*Micrococcus luteus* (CP001628; 99.6%)	39.1
		MZ339122	*Neobacillus niacini* (AB021194; 99.2%)	4.3
		MZ339123	*Rhodococcus yunnanensis* (BCXH01000047; 99.7%)	4.3
		MZ339124	*Streptomyces gardneri* (AB249908; 100%)	4.3
		MZ339125	*Streptomyces olivaceus* (JOFH01000101; 99.5%)	8.7

Supplementary Table S12. Continuation.

Sampling	CFU/m³ air	Accession Number	Identification (% identity)	Abundance (%)
Outdoor air	1190±300	MZ339126	*Arthrobacter bussei* (MN080869; 99.9%)	16.9
		MZ339127	*Arthrobacter gandavensis* (AJ316140; 99.9%)	3.4
		MZ339128	*Bacillus altitudinis* (ASJC01000029; 99.9%)	0.5
		MZ339129	*Bacillus aryabhattai* (EF114313; 100%)	2.3
		MZ339130	*Bacillus paramycoides* (MAOI01000012)/ *B. paranthracis* (MACE01000012)/ *B. nitratireducens* (KJ812430); (99.8%)	4.5
		MZ339131	*Bhargavaea beijingensis* (EF371374; 99.6%)	4.0
		MZ339132	*Bhargavaea ginsengi* (EF371375; 99.9%)	1.7
		MZ339133	*Brevibacterium frigoritolerans* (AM747813)/ *Peribacillus simplex* (BCVO01000086); (99.9%)	0.5
		MZ339134	*Citricoccus zhacaiensis* (EU305672; 98.7%)	2.8
		MZ339135	*Curtobacterium flaccumfaciens* (AJ312209; 100%)	11.3
		MZ339136	*Cytobacillus oceanisediminis* (GQ292772; 98.5%)	1.1
		MZ339137	*Exiguobacterium mexicanum* (AM072764; 100%)	8.4
		MZ339138	*Kocuria rosea* (X87756; 99.6%)	1.1
		MZ339139	*Microbacterium aurum* (CP018762; 98.4%)	6.2
		MZ339140	*Microbacterium endophyticum* (KJ920264; 98.5%)	10.7
		MZ339141	*Pseudarthrobacter equi* (LT629779; 100%)	7.4
		MZ339142	*Pseudarthrobacter phenanthrenivorans* (CP002379; 99.9%)	5.6
		MZ339143	*Rathayibacter caricis* (AF159364; 99.7%)	2.3
		MZ339144	*Rhodococcus cerastii* (FR714842; 99.7%)	2.3
		MZ339145	*Staphylococcus equorum* subsp. *equorum* (AB009939; 100%)	2.8
		MZ339146	*Streptomyces daghestanicus* (DQ442497)/ *S. albidoflavus* (Z76676)/ *S. violascens* (AY999737); (99.7%)	1.7
		MZ339147	*Streptomyces prasinus* (LIRH01000162; 98.3%)	2.3
